# Chloride Ion Transport Properties in Lightweight Ultra-High-Performance Concrete with Different Lightweight Aggregate Particle Sizes

**DOI:** 10.3390/ma15196626

**Published:** 2022-09-23

**Authors:** Yang Li, Gaozhan Zhang, Jun Yang, Yi Ding, Qingjun Ding, Yuxuan Wang

**Affiliations:** 1 Advanced Building Materials Key Laboratory of Anhui Province, Anhui Jianzhu University, Hefei 230022, China; 2 Anhui Survey & Design Institute of Water Resources & Hydropower Co., Ltd., Hefei 230001, China; 3 School of Material Science and Chemical Engineering, Anhui Jianzhu University, Hefei 230601, China; 4 State Key Laboratory of Silicate Materials for Architectures, Wuhan University of Technology, Wuhan 430070, China

**Keywords:** lightweight aggregate, ultra-high-performance concrete, particle size, chloride ion transport properties, numerical simulation

## Abstract

In this paper, the microstructure and resistance to chloride ion penetration of ultra-high-performance concrete (UHPC) prepared from lightweight aggregate (LWA) were studied through simulation and experiment. The effects of LWA with different particle sizes on the chloride ion transport properties of lightweight ultra-high-performance concrete (L-UHPC) were discussed through simulation test results. The random delivery model of LWA in L-UHPC was established by MATLAB, and the model was introduced into COMSOL. Through the comparative analysis of experimental data and simulation results, the repeatability of the proposed model and the simulation accuracy were verified. The results show that when the LWA particle size changes from 0.15–4.75 mm to 0.15–1.18 mm, the width of interfacial transition zone (ITZ) and the overall porosity of L-UHPC decrease. This is because the large particle size LWA has more open pores with larger pore diameters and related interconnections, which are potential channels for chloride ion transport. Therefore, the chloride ion transport properties in L-UHPC are inhibited, which is manifested by the “tortuosity effect” of the LWA.

## 1. Introduction

Reinforcement corrosion is one of the main reasons for the reduction in durability of reinforced concrete (RC) structures in service environment, and chloride contamination is the main factor leading to reinforcement corrosion [1]. Concrete is a three-phase system consisting of solid-phase mortar, aggregates, and liquid-phase water and gas-phase air. Liquid-phase water and gas-phase air are present in pores and cracks that provide channels for chloride ion transport [2]. Thus, reinforcement corrosion in RC is highly correlated with the porous nature of concrete [3,4,5,6]. As one of the important components of concrete, aggregate is usually dispersed in the concrete non-uniformly to form the skeleton of concrete, so aggregate directly affects the properties of concrete [7,8,9,10]. Though the traditional aggregate itself is relatively dense and chloride ions cannot be transported through it, the interfacial transition zone (ITZ) formed around the aggregate provides a channel for the transport of chloride ion [11]. The effect of aggregate on chloride ion transport can be attributed to the following four effects, namely dilution effect, tortuosity effect, ITZ and percolation effect [12]. When the free chloride ion concentration on the surface of steel bars exceeds 3.0%, the internal structure of RC will be destroyed, reducing the service life of RC [13]. At present, the methods of studying chloride ion transport in concrete are mainly macroscopic and mesoscopic. Macroscopic methods include the natural diffusion method and electric acceleration method. Mesoscopic methods are simulated by establishing concrete mesoscopic models [14,15]. Jin [16] studied the effect of coarse aggregate content on the chloride ion diffusion coefficient. It was found that with the increase in coarse aggregate content, the chloride ion diffusion coefficient gradually decreased. Based on experimental data, Xu [17] et al. performed mesoscopic finite element numerical simulation using Comsol, modeling concrete as a three-phase composite material consisting of mortar, aggregate and ITZ, and established a chloride ion simplified empirical model of long-term diffusion in concrete. Jin et al. [18] pointed out that the internal structure of concrete determines its durability, and studying its mechanism from the meso-scale can fundamentally improve the durability of concrete. Through the above studies, a deeper understanding of the diffusion simulation of chloride ions in ordinary concrete has been obtained, but there are still some problems. The COMSOL model needs to set parameters such as the chloride ion diffusion coefficient of the cement mortar and ITZ, while L-UHPC has a very low water-to-binder ratio and the optimized ITZ obtained by curing in PLWA. COMSOL has not yet been used to model chloride diffusion in L-UHPC. At the same time, since the LWA particle size is directly related to the internal curing effect, the numerical simulation of chloride ion diffusion for L-UHPC prepared with different LWA particle size ranges is different from other concretes.

Compared with traditional concrete, ultra-high-performance concrete (UHPC) has a denser structure, excellent durability, and can effectively resist the erosion of chloride ions in the environment, so it is widely used in bridge engineering, highway engineering, marine engineering, and military engineering [19,20,21]. As UHPC takes the closest packing as the core design principle, it has problems of self-heavy and large shrinkage, which limits its further promotion and application in the engineering field [22,23,24]. Lv [25] conducts chloride ion erosion experiments on UHPC samples with different pre-cracked degrees, and it is found that the content of free chloride ions has a great relationship with the initial damage of UHPC. Fan [26] conducted erosion experiments on UHPC with different crack widths and found that with the increase in crack width, the corrosion rate of steel bars increased. Ebru [27] believes that doing a good job in the prevention and control of UHPC shrinkage can effectively avoid the appearance of UHPC cracks, thereby reducing or even avoiding the corrosion of UHPC by chloride ions. Therefore, weight reduction and shrinkage reduction in UHPC are important directions for its development. While cement and steel fiber are the key factors to ensure the mechanical properties and durability of UHPC, the lightweight UHPC is realized from aggregate [28,29,30].

Our research group [31] used high-water-absorption lightweight aggregate (LWA) to prepare lightweight ultra-high-performance concrete (L-UHPC) with a surface density of less than 2000 kg/m^3^ and a shrinkage value of only 62.5% of that of traditional UHPC, which has excellent mechanical properties and volume stability. LWA achieves water release in the paste with reduced humidity in the later stage through pre-wetting treatment in the early stage. This unique internal curing mechanism can reduce or even avoid the shrinkage of L-UHPC, making the structure of L-UHPC more compact, thereby improving the resistance to chloride ion penetration. Compared with quartz sand, LWA has more pores and more complex ITZ; however, its influence mechanism on chloride ion transport in UHPC is unknown.

The aim of this work is to study the chloride ion transport properties of L-UHPC. To achieve this, the microstructure of the L-UHPC is first evaluated to characterize the internal environments for the chloride ion corrosion. Then, the corrosion behavior is monitored by using rapid chloride migration method (RCM) and electric flux method. The surface morphology and two-dimensional morphology of the ITZ of the LWA are characterized by using scanning electron microscopy (SEM). The width of ITZ is characterized by using energy-dispersive X-ray spectroscopy (EDS). The porosity of L-UHPC is tested by low-field nuclear magnetic resonance (LF-NMR). The capillary water absorption of L-UHPC is determined by the capillary water absorption method. Finally, the chloride ion transport properties in L-UHPC were investigated using Comsol simulation. This study provides a theoretical basis and technical support for the application of LWA in UHPC. Furthermore, a method is provided for the evaluation of the service life of L-UHPC structures in a chloride salt environment.

## 2. Experimental Program

### 2.1. Materials

The selected LWA must be able to achieve the core goal of “introducing internal curing water into the interior of L-UHPC”. The shale ceramic has been used and patented as an LWA since 1918. Shale ceramic not only has high water absorption but its flat shape is conducive to lap steel fibers and stabilize the paste. In this paper, the LWA is shale ceramic with a size ranging from 0.15 to 4.75 mm, with an apparent density of 1500 kg/m^3^ and a mass water absorption rate of 22.51% during saturated pre-wetting. In addition, the P·O 52.5 cement, silica fume (SF), and fly ash (FA) used in the present work were supplied by companies in mainland China. The compositions of the cement, SF, and FA obtained by X-ray fluorescence spectrometry (XRF) are shown in Table 1. The superplasticizer (SP) with a water-reducing rate of 30% was used to prepare the cement paste. In addition, brass-coated smooth steel fiber with dimensions of 13 mm length and 0.22 mm diameter, with a minimum tensile strength of 2850 MPa, was utilized.

### 2.2. Mix Proportion Design and Preparation of L-UHPC

#### 2.2.1. Mix Proportion Design

The composition of L-UHPC cementitious material is cement: SF: FA = 1:0.26:0.21; the particle size distribution of LWA is calculated by the modified Andreasen–Andersen method. The LWA particle sizes used in the test were 0.15–4.75 mm, 0.15–2.36 mm, and 0.15–1.18 mm. The mass ratio in each interval of the three LWA particle sizes is shown in Table 2.

Based on the above-mentioned particle gradation composition of cementitious material and LWA, the benchmark mix proportion of L-UHPC was determined by adjusting the binder-aggregate ratio and the admixtures, as shown in Table 3.

#### 2.2.2. Preparation and Curing of L-UHPC

Firstly, LWA was mixed according to particle gradation requirements and then put into mesh bags and immersed in water for pre-wetting. After the LWA was soaked for 24 h, the desired pre-wetted lightweight aggregate (PLWA) was obtained by paving it until the surface was dry. The cement, SF, and FA weighed according to the mix proportion were poured into the concrete mixer and mixed for 1 min. After the cementitious materials were evenly mixed, water and SP were added in and mixed for 2 min until a uniform flow was obtained. Then, the PLWA was added while stirring for 5 min, then molding and forming was performed. The surface of the forming mold was covered with plastic wrap and then placed in the standard curing box for one day to be taken out. After the mold was removed, the samples were maintained in the standard curing box until the specified age. The samples for resistance to chloride ion penetration were the standard samples specified in GB/T50082-2009 “Standards for Long-term Performance and Durability of Ordinary Concrete”.

#### 2.2.3. Basic Mechanical Properties of L-UHPC

The apparent density and working performance of L-UHPC were tested according to GB/T 50080-2016 “Standard for test method of performance on ordinary fresh concrete”. The strength of L-UHPC was tested according to the national standard GB/T 31387-2015 “Reactive powder concrete”. It can be observed from Table 4 that due to the apparent density of LWA being low, the apparent density of L-UHPC is less than 2000 kg/m^3^, and it decreases with the decrease in the LWA particle size. With the decrease in the LWA particle size, the slump and expansion of L-UHPC both increased first and then decreased. The compressive strength of the L-UHPC gradually increased with the decrease in the LWA particle size, and the flexural strength increased slightly. This is because the closest packing is used as the design principle of L-UHPC. The smaller the LWA particle size, the better the filling effect, and the overall porosity of L-UHPC is reduced. Therefore, the mechanical property of L-UHPC is improved, and the slump and expansion of L-UHPC are reduced.

### 2.3. Experimental Program

#### 2.3.1. Testing Method

(1) Anti-chloride ion penetration performance

**Figure 1:**
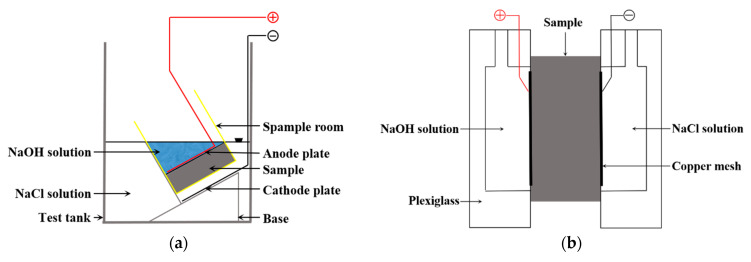
Schematic diagram of sample resistance to chloride ion penetration test; (**a**) electric flux method; (**b**) RCM.

According to GB/T50082-2009 “Standards for Long-term Performance and Durability Test of Ordinary Concrete”, the corrosion behavior was monitored by using rapid chloride migration method (RCM) and electric flux method. The specific test method is shown in Figure 1. Steel fibers were not incorporated in the test samples, and only the permeability of the cement matrix material was examined. The specific test method is shown in Table 5.

(2) Micromorphology

ZEISS GeminiSEM 500 SEM (Oberkochen, Germany) was used to observe the microscopic morphology of the samples. The low vacuum degree was 1–210 Pa, the voltage was 0.5–32 kV, the magnification was 18–300,000 times, and the resolution was 4.0 nm.

(3) The width of ITZ

EDS line scan is used to analyze the element distribution of LWA and cement paste to judge the width of the ITZ, as shown in Figure 1.

**Figure 2:**
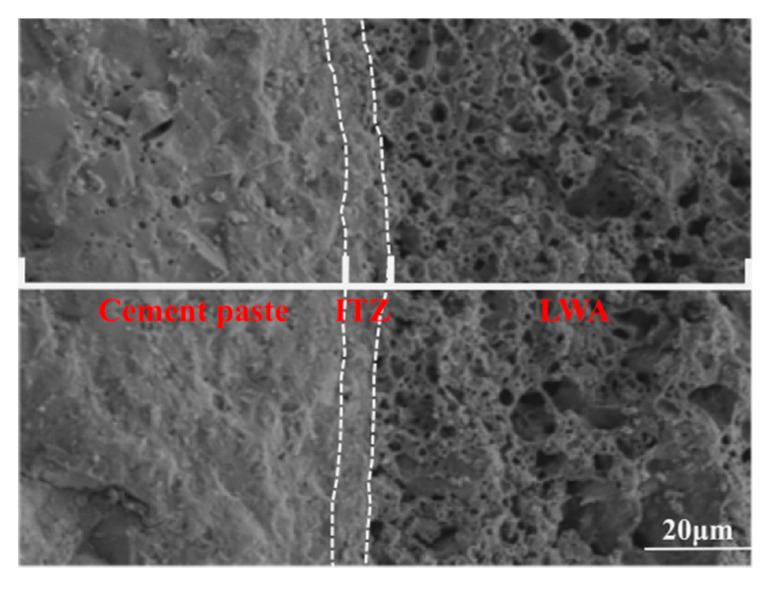

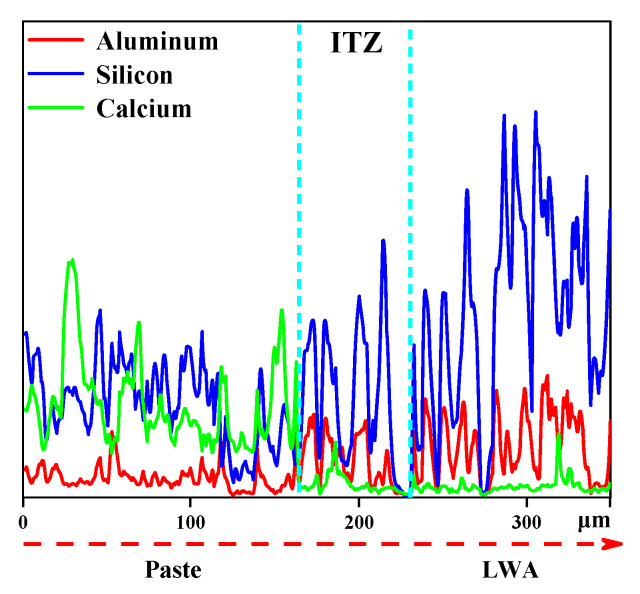
Interface element distribution.

Figure 1 shows the element distribution of LWA-ITZ-cement paste. The left side is the cement paste element distribution, the middle is the ITZ, and the right side is the LWA. It can be observed from Figure 2 that the Si concentrations were observed in the ITZ similar to that in the paste but was lower than in the LWA; the Ca element is mainly distributed in the paste; the Al element is mainly distributed in the LWA, less in the paste and the ITZ. Therefore, the ITZ width of LWA can be calculated by Si, Ca and Al elements [28,32,33].

(4) Microhardness

For this experiment, a DHV-1000 microhardness tester was used to test the microhardness value of L-UHPC. After preparing the 40 × 40 × 40 mm samples, after curing to the age, a precision cutting machine was used to cut the samples, and the middle width of the samples with a thickness of 1 cm was selected, which were put in an alcohol solution for immersion and saved, then taken out for drying before testing. During the test, the LWA was taken as the first test point, and then one point was taken every 20 μm for testing, and a total of 10 points were taken. This was repeated 10 times and the average value was taken as the data value for each test point.

(5) Pore

For this experiment, according to the ASTM C1585-2013 standard, the capillary water absorption of L-UHPC was determined by the capillary water absorption method. The MesoMR23-60 H medium-sized MRI analyzer (Niumag, Suzhou, China) was used to test the pore structure of the L-UHPC. Samples of 40 × 40 × 40 mm were used for testing. When the sample reached the curing age, it was soaked in alcohol and dried before the test. Before the LF-NMR test, samples were treated for water retention.

#### 2.3.2. Model and Parameters

**Figure 3:**
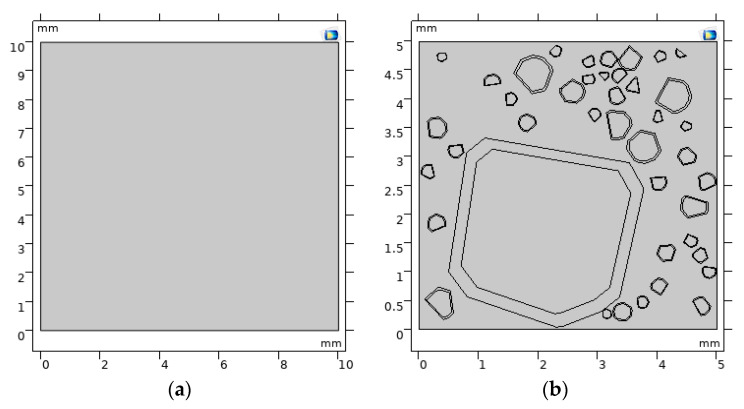
L-UHPC model for LWA particle size of 0.15–4.75 mm. (**a**) Global model; (**b**) random aggregate model.

In this paper, an L-UHPC global model and a two-dimensional three-item random aggregate model are established, wherein the three items are cement paste, LWA and ITZ, as shown in Figure 3. Figure 3a is a two-dimensional L-UHPC global model, and Figure 2b is a two-dimensional three-term L-UHPC random aggregate model. The volume fraction of the aggregate is 42%, and the shape of the aggregate is a polygon with 8–12 sides. Each LWA is evenly wrapped with a layer of ITZ, and the thickness of the ITZ is set to be 0.15 times the LWA particle size. 

The proportion of each particle size is based on the design in Section 2.2.1, and the positions are randomly generated by MATLAB.

In this paper, the chloride ion transport simulation of L-UHPC simulation is carried out by using the dilute material transport plate in the COMSOL. Based on the experimental data and related literature [34,35,36,37,38,39], the specific parameters are shown in Table 6, Table 7 and Table 8.

## 3. Results and Discussion

### 3.1. Chloride Ion Transport Properties

**Figure 4:**
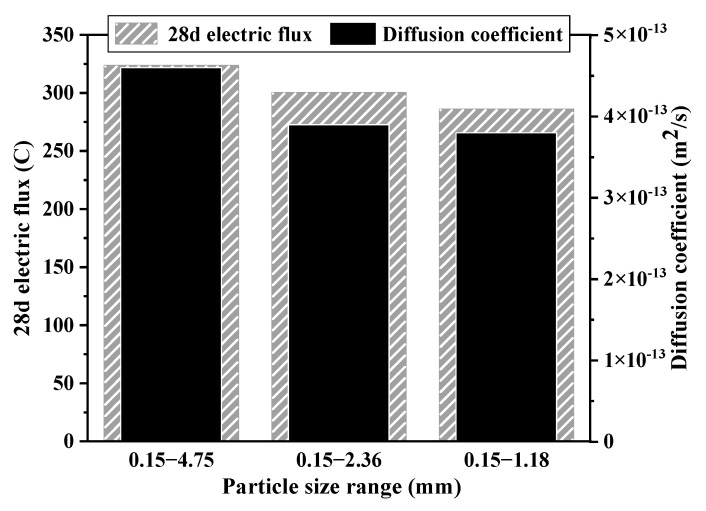
Effect of the LWA particle size on the chloride ion transport properties in L-UHPC.

Figure 4 shows the effect of the LWA particle size on the chloride ion transport properties in L-UHPC. It can be observed from Figure 3 that with the increase in the LWA particle size, the chloride ion diffusion coefficient of L-UHPC gradually increases, indicating that the LWA particle size has a great impact on the chloride ion transport properties of L-UHPC. Compared with traditional UHPC whose chloride ion diffusion coefficient is 0.69 × 10^−12^ m^2^/s and electric flux range is 400 C [40], when using 0.15–1.18 mm LWA, the chloride ion diffusion coefficient and electric flux of L-UHPC decreased by 44.9% and 28.5%, respectively.

**Figure 5:**
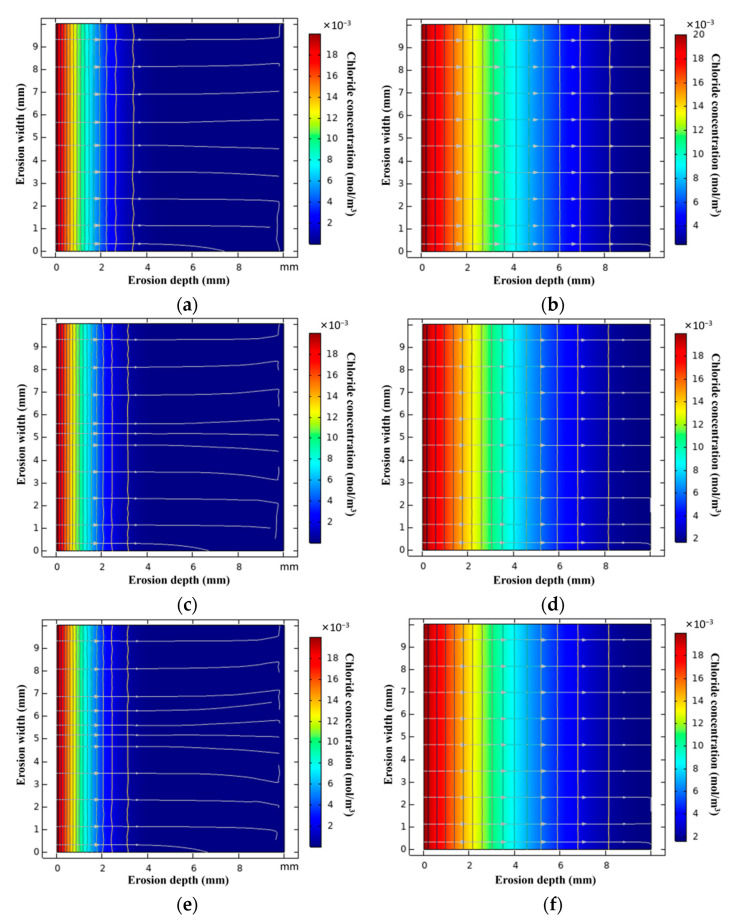
(**a**) 0.15–4.75 mm–30 d; (**b**) 0.15–4.75 mm–360 d; (**c**) 0.15–2.36 mm–30 d; (**d**) 0.15–2.36 mm–360 d; (**e**) 0.15–1.18 mm–30 d; (**f**) 0.15–1.18 mm–360 d.

**Figure 6:**
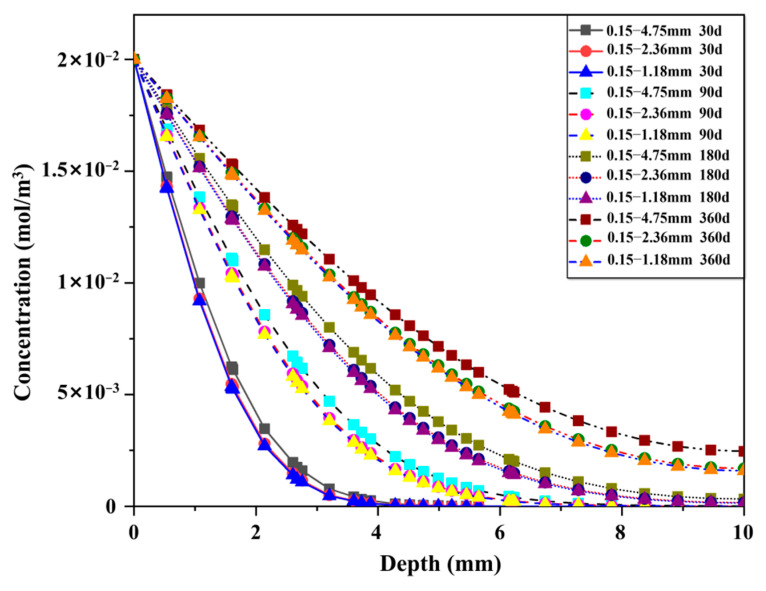
Effect of LWA particle size on chloride ion transport in L-UHPC under different erosion age.

Figure 5 shows the distribution of chloride ions in L-UHPC after chloride ion erosion for 30 d and 360 d in the simulated state. Figure 6 shows the effect of LWA particle size on the chloride ion transport of L-UHPC at 30 d, 90 d, 180 d, and 360 d erosion ages. From the analysis of Figure 4 and Figure 5, it can be observed that the erosion depth of chloride ions gradually decreases with the reduction in the LWA particle size. This is due to the following reasons: (1) the reduced LWA particle size improves the internal compactness of L-UHPC, which reduces the total porosity and the chloride ion transmission channel of L-UHPC; (2) with the reduction in the LWA particle size, the internal curing effect of the LWA is more prominent, which optimizes the structure of ITZ around the LWA and hinders the transmission of chloride ions. When the erosion period is 30 d, the erosion depth of L-UHPC is only about 4 mm; after 360 d erosion, the erosion depth is more than 10 mm. This is consistent with the variation trend of transmission depth in the UHPC seawater splash zone in the study of Ma [41]. Additionally, this is attributed to the increase in erosion age; chloride ions react with hydrated calcium aluminate and Ca(OH)_2_ in the ITZ, resulting in the increase in cracks and pores in the ITZ [42,43], which further accelerates the transport rate of chloride ions in L-UHPC. Moreover, it can be seen from the analysis of Figure 6 that with the increase in the erosion time, the transmission rate of chloride ions of the specimen with a larger LWA particle size is significantly faster than that of the specimen with a smaller LWA particle size. This is because the pore size and porosity of LWA is proportional to its particle size; when the pore size of LWA increases, the chloride ion transport channel increases. Therefore, with the increase in LWA particle size, the internal structure of ITZ is more deteriorated.

### 3.2. ITZ

**Figure 7:**
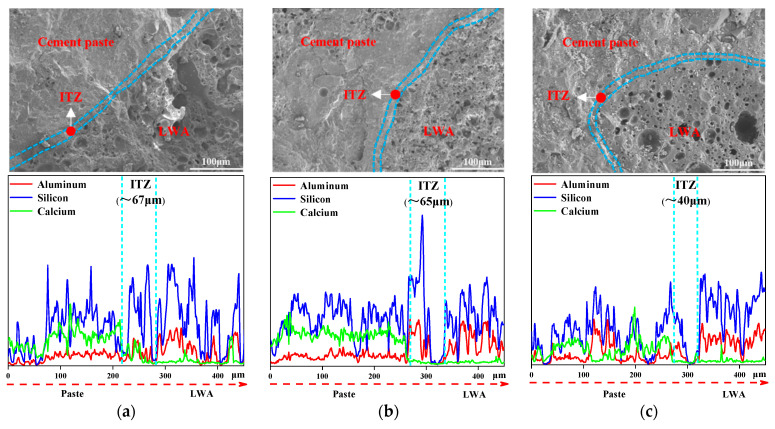
ITZ prepared by LWA with different particle sizes: (**a**) 0.15–4.75 mm; (**b**) 0.15–2.36 mm; (**c**) 0.15–1.18 mm.

The morphologies of the ITZ of L-UHPC with particle sizes of 0.15–4.75 mm, 0.15–2.36 mm, and 0.15–1.18 mm for the LWA are shown in Figure 7. It can be observed from Figure 7 that the widths of the ITZ of the L-UHPC with particle sizes of 0.15–4.75 mm, 0.15–2.36 mm, and 0.15–1.18 mm for the LWA are 67 μm, 65 μm, and 40 μm, respectively. With the decrease in the LWA particle size, the LWA and the hardened cement paste are closely combined, and there is no obvious interface. This is because the microstructure of the ITZ between the cement paste and the LWA depends largely on the properties of the LWA. Compared with the porous ITZ around the normal weight aggregate, LWA has no wall effect, and the microstructure of the ITZ formed on its surface is comparable to that of the cement paste [44,45,46,47]. Additionally, the surface of the LWA is mostly open pores, which can tightly wrap the cement paste [48]. During the curing process of concrete, the free water of PLWA is slowly released, and the interface area of LWA-cement paste is fully hydrated. Therefore, the ITZ is improved, and the tortuosity of the chloride ion transport path is increased. The LWA surface is rough and has open pores. When the LWA particle size is large, there are more open pores and connected pores on the surface of LWA, the water release rate increases, and the impact range is large, so the width of ITZ is large. The opposite is true when the LWA particle size is small.

**Figure 8:**
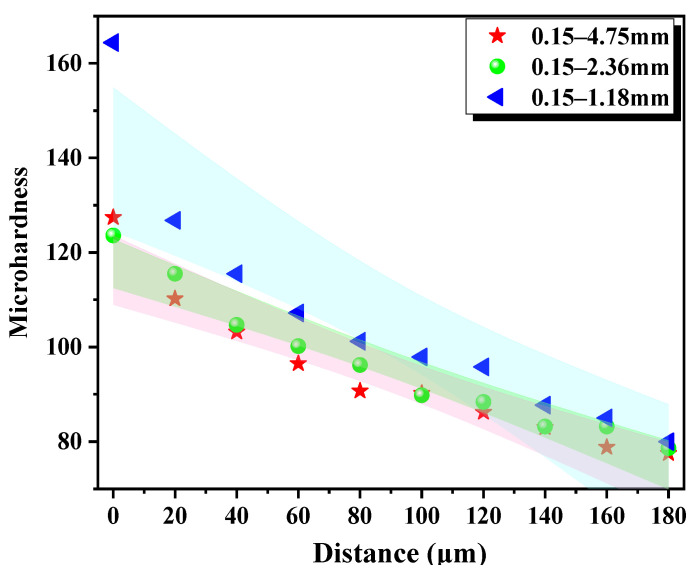
Microhardness of LWA-ITZ-hardened cement paste of LWA with different particle sizes. Colored bands represent 95% confidence band.

It can be observed from Figure 7 and Figure 8 that the LWA particle size is proportional to the ITZ width and inversely proportional to the microhardness value. It can be attributed to the openings on the surface of the LWA being large; the pre-wetted water will be released in advance, resulting in the deterioration of the ITZ. In the later curing, the water release rate is faster, and a water film is formed around the LWA, which causes the deterioration of the ITZ. This is not good for L-UHPC to resist the invasion of chloride ions [49,50]. When chloride ions migrate to the ITZ, they react with hydrated calcium aluminate and Ca(OH)_2_ in the ITZ, which further leads to the increase in microcracks in the ITZ, providing migration channels for chloride ions. Therefore, the LWA particle size has an influence on the resistance to chloride ion penetration of L-UHPC.

### 3.3. Pore

**Figure 9:**
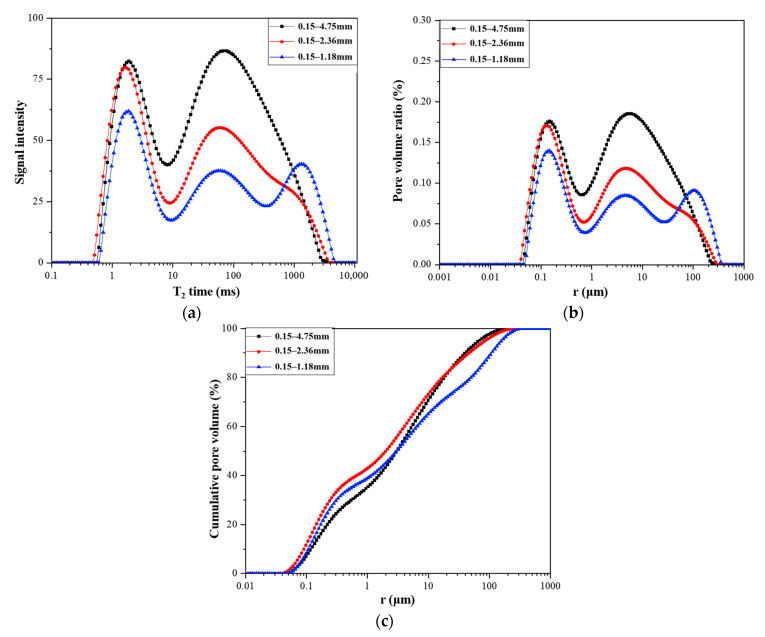
Effect of LWA particle size on the pore structure of L-UHPC. (**a**) Effect of LWA particle size on the T_2_ spectra of L-UHPC; (**b**) effect of LWA particle size on the pore size distribution of L-UHPC; (**c**) effect of LWA particle size on the cumulative pore size distribution of L-UHPC.

**Figure 10:**
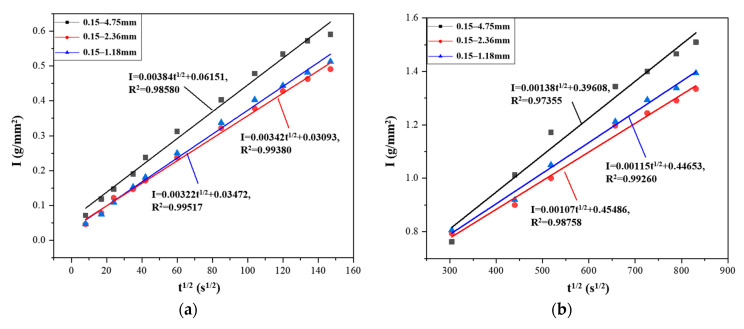
Effect of LWA with different particle sizes on water adsorption of L-UHPC; (**a**) initial adsorption; (**b**) secondary adsorption.

PLWA provides additional water for the continuous hydration of cement in L-UHPC and the pozzolanic reaction of auxiliary cementitious materials, so ITZ is enhanced, which reduces the internal porosity and permeability of L-UHPC [51,52]. Meng [53] reported that the partial replacement of river sand with pre-wet fine sand in UHPC can effectively reduce the total porosity of UHPC. Pores are an important channel for chloride ion transport in L-UHPC. The pores in L-UHPC can be divided into two types: one is the pores of the LWA itself, and the other is the pores in the hardened cement paste. Generally speaking, the pore size range in cement paste is smaller than that of LWA [54]. It can be observed from Figure 9 that reducing the LWA particle size not only reduces the content of small pores and macropores but also accelerates the accumulation rate of small pores, effectively refines the internal pore structure of L-UHPC, and reduces the total porosity of concrete. Moreover, it can be observed from Figure 10 that reducing the LWA particle size reduces the primary and secondary adsorption coefficient of L-UHPC and effectively improves the capillary structure of L-UHPC. Liu [55] reported that under the same LWA content, the incorporation of LWA with a smaller particle size into ultra-high-strength concrete (UHSC) could increase the volume of protective slurry, thereby reducing the permeability of UHSC.

**Figure 11:**
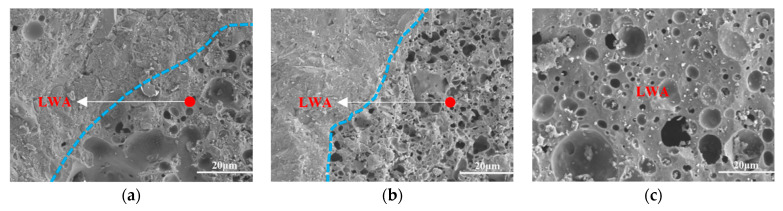
SEM images of LWA with different particle sizes: (**a**) 0.15–4.75 mm; (**b**) 0.15–2.36 mm; (**c**) 0.15–1.18 mm.

LWA have more pores, which can be divided into open pores and closed pores. Closed pores are relatively isolated and not connected to each other, which can effectively block the transmission path of chloride ions; however, open pores are usually larger in diameter and connected to each other, which may become potential channels for chloride ion transmission [15]. It can be observed from Figure 11 that the larger the LWA particle size, the more connected pores on the surface and inside the LWA, which provides a potential channel for chloride ion transport. In addition, the larger the LWA particle size, the more pores on the LWA surface. This leads to the fact that during the curing process of L-UHPC, the cementitious paste enters the interior of the LWA, and a hydration product C-S-H gel is formed at a special site, which can effectively improve the structure of the LWA and the ITZ. Additionally, it was also found that the larger the LWA particle size, the more C-S-H gel generated inside the LWA, and vice versa. When the LWA particle size is 0.15–1.18 mm, the pore structure inside the LWA is mostly isolated pores, which can block the transmission path of chloride ions.

**Figure 12:**
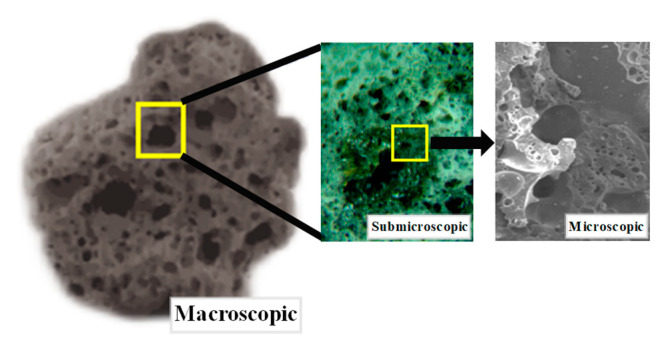
Morphology of LWA.

In conclusion, reducing the LWA particle size can refine the pore structure of L-UHPC, reduce the total porosity, and reduce the migration channel of chloride ions; in addition, as shown in Figure 12, for the pores of LWA itself, under the same pre-wetting condition, the water inside the LWA also becomes a diffusion medium for chloride ions, and it provides a certain potential channel for chloride ion transmission. However, for LWA with a small particle size, the pores are mostly isolated closed pores, which further hinders the transmission of chloride ions to a certain extent.

### 3.4. Influence Mechanism

The migration of chloride ions mainly takes water as the medium, enters the interior through the surface defects of the concrete, and then utilizes the internal pores and micro-cracks in the concrete as the transmission channel for internal migration. For the porous and “fragile” ITZ, the transmission rate of chloride ions in it is about 6–12 times higher than that in cement paste [36,37]. Therefore, the ITZ and pore structure characteristics are the key factors affecting chloride ion transport.

#### 3.4.1. Macro-Scale

Table 9 shows the relevant parameters of L-UHPC with different particle sizes measured by each test method. It can be observed from Table 8 that as the maximum LWA particle size increased from 1.18 mm to 4.75 mm, the diffusion coefficient of L-UHPC increased by 21%, the width of the ITZ increased by 67.5%, the porosity increased by 62.8%, and the adsorption coefficient increased by 19.3%.

From the analysis of Table 9, it can be observed that there were more openings on the surface with the increase in LWA particle size, which leads to the early loss of pre-wet moisture during the mixing process and deteriorates the L-UHPC. Not only does this increase the total porosity, the increase in capillary pores results in a coherent network of pores [55]. In addition, the increase in the LWA particle size also has a negative impact on the ITZ. The larger particle size of LWA releases water faster to form a water film on the surface, which increases the width of the ITZ and increases the cracks and pores in the ITZ [56]. Therefore, when chloride ions enter the interior of L-UHPC, the pores in the matrix serve as the transport channels, and the pores and cracks in the ITZ further expand the transport channels and accelerate the transport rate of chloride ions in L-UHPC. Reducing the LWA particle size can reduce the number and area of pores on the surface of the LWA, thus effectively reducing the negative impact of the mixing process and curing process. As a result, the density of the L-UHPC is improved, the width of the ITZ is narrower, and the strength is higher. Therefore, when chloride ions are transported inside L-UHPC, there are fewer pores in the matrix and fewer chloride ion transport channels, and the ITZ is no longer a weak region. Not only that, when the LWA particle size is small, the interior is made up of mostly closed and independent solitary pores, which can further hinder the transmission of chloride ions in L-UHPC. This is consistent with the variation law of compressive strength.

#### 3.4.2. Meso-Scale

**Figure 13:**
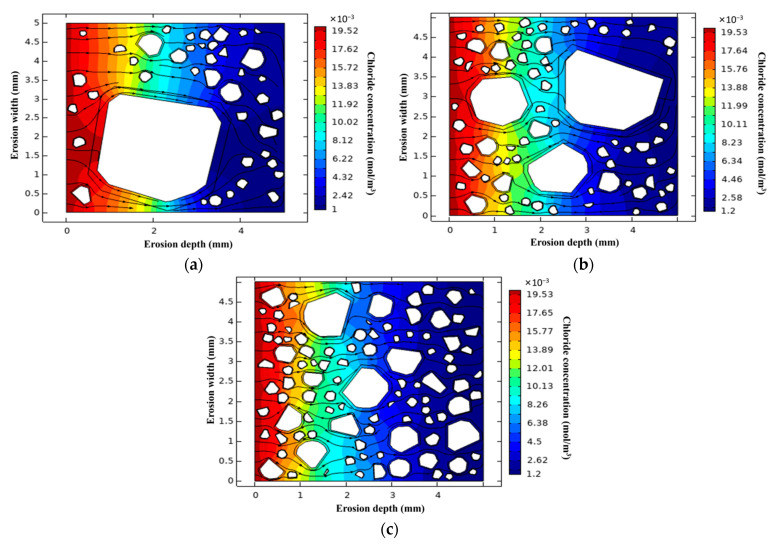
Simulation results of L-UHPC random aggregate model of LWA with different particle sizes: (**a**) 0.15–4.75 mm; (**b**) 0.15–2.36 mm; (**c**) 0.15–1.18 mm.

The simulation results of the L-UHPC random aggregate model prepared by LWA with different particle sizes are shown in Figure 13. Due to D_ITZ_ being larger than D_cp_, the volume fraction of ITZ increases with the increase in LWA particle size, which provides a channel for the rapid transmission of chloride ions. Furthermore, when the volume fraction of LWA is the same, the number of LWA gradually increases with the decrease in LWA particle size, which increases the tortuosity inside the L-UHPC. In the same region, when chloride ions enter the interior of the L-UHPC through surface pores or microcracks, the increased tortuosity of the LWA lengthens the transport path of the L-UHPC. As the transmission distance increases, the chloride ion concentration per unit volume is continuously diluted, and the chloride ion transmission rate continue to decrease [54]. The LWA particle size is reduced, which can effectively hinder the transmission of chloride ions.

## 4. Conclusions

In this paper, the effect of the light-aggregate particle size on the microstructure and resistance to chloride ion penetration of L-UHPC was found through indoor immersion and simulation tests. The microstructure of L-UHPC is directly related to its resistance to chloride ion penetration. COMSOL was used as an efficient simulation method to study the long-term resistance to chloride ion permeation of L-UHPC. From the experimental results, the following conclusions can be drawn:

1. With the reduction in the particle size range of LWA, the working performance of L-UHPC shows a trend of first improving and then weakening. When the particle size of the LWA is in the range of 0.15–2.36 mm, L-UHPC has the best working performance, and its expansion can reach 550 mm.
2. The LWA particle size determines the ITZ structure of L-UHPC. When the maximum particle size of LWA decreased from 4.75 mm to 1.18 mm, the width of ITZ gradually decreased, and the microhardness gradually increased. The denser ITZ played a positive role in hindering the transport of chloride ions.
3. LWA with a smaller particle size can refine the L-UHPC pore structure more effectively. As the LWA particle size decreased from 0.15–4.75 mm to 0.15–1.18 mm, the number of capillary pores inside L-UHPC decreased, the number of self-isolated pores increased, and the total porosity decreased from 12.96% to 7.96%. The reduction in pores reduces the chloride ion transport channels, thereby improving the resistance of L-UHPC to chloride ion penetration.
4. The decrease in LWA particle size increases the internal tortuosity of L-UHPC. Since the small particle size of LWA prolongs the transmission path of chloride ions, the chloride ions are continuously diluted when they are transported inward, resulting in a gradual decrease in the migration rate of chloride ions in L-UHPC. Therefore, with the decrease in LWA particle size, the resistance to chloride ion penetration of L-UHPC gradually increased. When the particle size of LWA is 0.15–1.18 mm, the chloride ion diffusion coefficient of L-UHPC is only 0.38 × 10^−12^ m^2^/s.

## Figures and Tables

**Table 1:** Chemical composition of cementitious materials (wt%).

Material	SiO_2_	Al_2_O_3_	Fe_2_O_3_	CaO	MgO	Na_2_O	SO_3_	Loss on Ignition
Cement	21.10	6.16	4.41	64.80	3.28	0.48	2.06	1.78
SF	93	1.00	0.70	0.50	0.70	0.08	0.15	3.10
FA	52.00	22.00	4.00	12.00	0.56	0.43	0.35	1.00

**Table 2:** The mass proportion of each interval of LWA (%).

Particle Size (mm)	Interval of the Particle Size (mm)				
	2.36–4.75	1.18–2.36	0.6–1.18	0.3–0.6	0.15–0.3
0.15–4.75 mm	27	23	19	17	14
0.15–2.36 mm	-	32	26	23	19
0.15–1.18 mm	-	-	38	33	29

**Table 3:** Benchmark mix proportion of L-UHPC (kg/m^3^).

Cement	SF	FA	LWA	Water
821	208	175	675	222

**Table 4:** Basic mechanical properties of L-UHPC.

LWA Particle Size (mm)	Slump (mm)	Expansion (mm)	Apparent Density (kg/m^3^)	Mechanical Property (MPa)	
				Flexural Strength	Compressive Strength
0.15–4.75	220	430	1980	18.9	101.2
0.15–2.36	250	550	1960	19.6	110
0.15–1.18	249	470	1870	20.1	126.7

**Table 5:** Test methods for resistance to chloride ion penetration.

Method	Experimental Conditions				Judging Parameters	Judgment Criteria
	Voltage	Anode Tank	Cathode Tank	Power-on Time		
Electric flux method	60 V	0.3 mol/L NaOH	3% NaCl	6 h	Electric flux (C)	>4000 C, high permeability; 2000–4000 C is medium; 1000–2000 C is low; 100–1000 C is very low; <100 C is negligible
RCM	30 V	0.3 mol/L NaOH	10% NaCl	Initial current determination time	Diffusion coefficient (cm^2^/s)	-

**Table 6:** Groups of global model parameters.

LWA Particle Size (mm)	L-UHPC Apparent Density (kg/m^3^)	LWA Density (kg/m^3^)	Chloride Diffusion Coefficient (×10^−12^ m^2^/s)	28 d Electric Flux (C)
0.15–4.75	1980	1630	0.46	324
0.15–2.36	1960	1670	0.39	300
0.15–1.18	1870	1770	0.38	286

**Table 7:** Random aggregate model parameter settings.

Group	D_cp_ *	D_ITZ_ **	D_LWA_ ***
Model parameter settings	1.64 × 10^−12^ m^2^/s	0.82 × 10^−12^ m^2^/s	0 × 10^−12^ m^2^/s

* D_cp_ is the chloride diffusion coefficient of the paste; ** D_ITZ_ is the chloride diffusion coefficient of the ITZ; *** D_LWA_ is the chloride diffusion coefficient of the LWA.

**Table 8:** Parameters related to chloride diffusion.

Related Parameters	Value
Boundary conditions	C(x,t) = 0.02 mol/m^3^
Initial conditions	C(x,0) = 0.00 mol/m^3^
Global erosion age	30 d, 90 d, 180 d, 360 d
Random aggregate model erosion age	30 d

**Table 9:** Relevant parameters of L-UHPC under different LWA particle sizes.

Group	0.15–4.75 mm	0.15–2.36 mm	0.15–1.18 mm
Diffusion coefficient (×10^−12^ m^2^/s)	0.46	0.39	0.38
ITZ width (μm)	67	65	40
Porosity (%)	12.96	9.65	7.96
Adsorption coefficient (g/(mm^2^·s^1/2^))	0.00384	0.00342	0.00322
28d compressive strength (MPa)	101.2	110	126.7

## Data Availability

Data is contained within the article.
